# When Chronic Conditions Become Acute: Prevention and Control of Chronic Diseases and Adverse Health Outcomes During Natural Disasters

**Published:** 2005-10-15

**Authors:** George A Mensah, Ali H Mokdad, Samuel F Posner, Eddie Reed, Eduardo J Simoes, Michael M Engelgau, Chronic Diseases and Vulnerable Populations in Natural Disasters Working Group

**Affiliations:** National Center for Chronic Disease Prevention and Health Promotion, Centers for Disease Control and Prevention; Division of Adult and Community Health, National Center for Chronic Disease Prevention and Health Promotion (NCCDPHP), Coordinating Center for Health Promotion (CoCHP), Centers for Disease Control and Prevention (CDC), Atlanta, Ga; NCCDPHP, CoCHP, CDC, Atlanta, Ga; NCCDPHP, CoCHP, CDC, Atlanta, Ga; NCCDPHP, CoCHP, CDC, Atlanta, Ga; Division of Diabetes Translation, NCCDPHP, CoCHP, CDC, Atlanta, Ga

Natural disasters pose major public health challenges. Preparations for these disasters usually focus on how to evacuate people from affected areas; how to provide transportation, shelter, food, and water for the evacuees; and how to prevent injury and infectious diseases that may develop in crowded living situations after disasters (1-3). All of these preparations are important and necessary, but they are not enough.

Also needed are preparations to care for populations whose health is already compromised and who are, therefore, more vulnerable than healthy people to the stresses and disruptions caused by natural disasters. Populations affected by disasters may carry a large and measurable burden of disabilities and chronic diseases, especially heart disease, cancer, stroke, diabetes, and chronic respiratory disorders (4). For example, we can reasonably project that the number of people with cancer who were directly affected by the Hurricane Katrina evacuation is in the tens of thousands (J. King, written communication, September 2005). In addition, data from the 2000 U.S. census long-form survey indicate that 20.2% of the population, roughly 275,000 people, aged 5 years and older in the 10 parishes in the greater New Orleans metropolitan area had a disability of some type (5). Accommodating the needs of this population during evacuation and return is a sizable undertaking.

Chronic illnesses are exacerbated by the conditions caused by a disaster (e.g., lack of food, lack of clean water, extremes of cold or heat, physical and mental stress, injury, exposure to infection). Natural disasters may also put people with limited mobility and women who are pregnant and their unborn fetuses at increased risk for adverse health outcomes. Elderly men and women, many of whom have multiple chronic conditions and comorbidities being treated with multiple medications, are particularly at risk (6-9). People of low socioeconomic status, people without health insurance, and people with mental illness or disabilities are other vulnerable populations who can experience higher morbidity and mortality during disasters. Similarly vulnerable are ischemic stroke survivors taking anticoagulants, people whose diabetes is controlled by insulin, heart attack survivors taking clot-preventing medications, people with severe lung disease receiving home oxygen therapy, people with hereditary blood disorders, and patients receiving hemodialysis for kidney failure (9-14).

Lack of access to routine health care is a leading cause of mortality after disasters (15). In addition, indirect effects (e.g., loss of electricity) can lead to exposure to extreme heat or cold or interruption of supplemental oxygen supplies. Many people living with disabilities rely on routine health care services to maintain their quality of life and live independently. Without access to these services, they may experience adverse health events. Compared with other pregnant women, women in the early stages of pregnancy may be at higher risk for adverse pregnancy outcomes because of exposure during organogenesis to toxins or infectious agents. Unfortunately the problems of vulnerable populations who are at risk for adverse health outcomes when routine health care services are disrupted remain inadequately studied or addressed.

The aftermath of Hurricane Katrina is a reminder of the urgent need for developing and implementing recommendations for the control of chronic diseases during disasters. A limited needs assessment among individuals staying in evacuation centers, conducted in the field and reported to the Centers for Disease Control and Prevention (CDC), demonstrated that five of the top six conditions were all chronic diseases and that, other than injuries, the majority of medical and health visits were for medication refills, oral health issues, and other chronic health conditions (16). Leading the list of top 10 conditions were hypertension and cardiovascular diseases, diabetes, and psychiatric disorders (new and existing) (16). Other surveys of Hurricane Katrina evacuees show that up to 41% had at least one chronic health condition such as heart disease, hypertension, diabetes, or asthma (17). The preliminary medical examiner report of mortality associated with Hurricane Charley in Florida in 2004 showed that the six deaths related to natural causes resulted from exacerbation of existing cardiac or pulmonary conditions (18). The editorial accompanying the report called for strengthening local disaster plans and public health messages for vulnerable populations who are likely to have chronic medical conditions and are likely to require medical supplies or equipment that depend on electricity to operate (18).

Preparations for the prevention and control of chronic diseases, of secondary conditions among people with disabilities, and of adverse pregnancy outcomes during disasters must be guided by 1) the predisaster rates of adverse health outcomes and disease burden, 2) awareness of the immediate needs of people with chronic diseases (including a plan for providing essential medications), 3) knowledge of the basic and surge capacity of health care delivery systems of the affected and surrounding areas to treat and manage chronic diseases, and 4) the areas' ability to rebuild the basic infrastructure needed to support care. A comprehensive strategy to address the overall health of disaster survivors must therefore include not only a plan for evacuation and emergency treatment but also a strategy to deliver care to vulnerable populations including pregnant women and people with chronic diseases or disabilities.

In accordance with established clinical and preventive services guidelines, disaster preparations must ensure the availability of everything necessary to control chronic diseases, prevent acute events and complications related to chronic diseases, and protect the health and well-being of pregnant women and their fetuses. These guidelines should address patient triage, clinical evaluation, and supply of essential medications for care of chronic illnesses (19-21). Support of the emergency medical response system and access to specialty care such as hemodialysis, ventilatory support, and emergency obstetric care must be delineated. A list of essential medications consistent with the predicted burden of chronic diseases should be developed and used in planning for provision of chronic maintenance medications during disasters.

The CDC, in partnership with the public health community, should consider developing surveillance tools to support disaster planning that adequately addresses the health care needs of the general population and of vulnerable populations, including pregnant women and people living with disabilities. Such a surveillance tool should have at least three components: 1) the ability to establish a baseline of the size, functional status, and needs of the vulnerable populations in areas susceptible to predictable disasters (such as hurricanes); 2) the ability to assess the needs and levels of actual response during the disasters; and 3) the ability to monitor the long-term effects of the emergency. These components map the surveillance activities into a timeline reflecting the three phases of the disaster: before, during, and after.

A wide range of professionals, including physicians, nurses, public health professionals, legislators, the aging services network, and national and community organizations must use the data generated from these assessments to guide policy development and to ensure the use of best practices during disasters. Appropriate policies should be established to support the development of training materials for health professionals so that they can care for vulnerable populations. Similar training materials could be used in medical schools, nursing schools, and public health institutions and as part of emergency relief training. A response to chronic diseases and obstetric health care needs during emergencies based on appropriate policies and best practices should be included in the emergency response plans of states and municipalities, and the planned response should be evaluated periodically by all the groups, agencies, and institutions involved in disaster planning. Public education materials, including public service announcements in affected areas, should remind people of routine steps needed to ensure that their chronic diseases remain stable and adverse health outcomes are prevented throughout a disaster. For example, in response to Hurricane Katrina, the CDC developed materials, including radio and television public service announcements, to deliver public health messages for pregnant women. These messages have been aired in multiple areas with large populations of evacuees. Messages were also developed for topics such as hand washing and were widely used in evacuee shelters. 

Although individual patients and their families need to be well prepared and provided with clear and consistent recommendations to make preparations, many others must help them. To ensure an adequate response, disaster preparation should be coordinated with all partners, roles should be well defined, and procedures should be clearly stated. Responsibilities for each element of the response should be assigned in advance. An adequate means of communication and standardized health procedures should be available.

The lessons of Hurricane Katrina should stimulate action long overdue to consider the importance of chronic diseases in disaster planning. It is time to carefully reflect on the chronic health needs of all populations and the health conditions that may be exacerbated during natural disasters. Although reducing the potential for infectious disease outbreaks is vital, minimum standards should be set to prevent and control morbidity and mortality among people with chronic diseases, people who are pregnant, and people with disabilities whose safety and quality of life may be adversely affected by a stressful interruption in their routine health care. It is time to develop and implement guidelines for both short- and long-term care before, during, and in the immediate aftermath of natural disasters.
